# Automatic identification of Parkinsonism using clinical multi-contrast brain MRI: a large self-supervised vision foundation model strategy

**DOI:** 10.1016/j.ebiom.2025.105773

**Published:** 2025-05-27

**Authors:** Xueling Suo, Mengyao Chen, Li Chen, Chunyan Luo, Graham J. Kemp, Su Lui, Huaiqiang Sun

**Affiliations:** a Department of Radiology, Institution of Radiology and Medical Imaging, West China Hospital of Sichuan University, Chengdu, 610041, Sichuan, China; b Functional and Molecular Imaging Key Laboratory of Sichuan Province, West China Hospital of Sichuan University, Chengdu, 610041, Sichuan, China; c Research Unit of Psychoradiology, Chinese Academy of Medical Sciences, Chengdu, 610041, Sichuan, China; d Department of Neurology, West China Hospital of Sichuan University, Chengdu, 610041, Sichuan, China; e Liverpool Magnetic Resonance Imaging Centre (LiMRIC) and Institute of Life Course and Medical Sciences, University of Liverpool, Liverpool, L69 3GE, United Kingdom

**Keywords:** Parkinson’s disease, Parkinson-plus syndrome, Self-supervised learning, Neuroimaging, MRI, Screening

## Abstract

**Background:**

Valid non-invasive biomarkers for Parkinson’s disease (PD) and Parkinson-plus syndrome (PPS) are urgently needed. Based on our recent self-supervised vision foundation model the Shift Window UNET TRansformer (Swin UNETR), which uses clinical multi-contrast whole brain MRI, we aimed to develop an efficient and practical model (‘SwinClassifier’) for the discrimination of PD vs PPS using routine clinical MRI scans.

**Methods:**

We used 75,861 clinical head MRI scans including T1-weighted, T2-weighted and fluid attenuated inversion recovery imaging as a pre-training dataset to develop a foundation model, using self-supervised learning with a cross-contrast context recovery task. Then clinical head MRI scans from n = 1992 participants with PD and n = 1989 participants with PPS were used as a downstream PD vs PPS classification dataset. We then assessed SwinClassifier’s performance in confusion matrices compared to a comparative self-supervised vanilla Vision Transformer (ViT) autoencoder (‘ViTClassifier’), and to two convolutional neural networks (DenseNet121 and ResNet50) trained from scratch.

**Findings:**

SwinClassifier showed very good performance (F1 score 0.83, 95% confidence interval [CI] [0.79–0.87], AUC 0.89) in PD vs PPS discrimination in independent test datasets (n = 173 participants with PD and n = 165 participants with PPS). This self-supervised classifier with pretrained weights outperformed the ViTClassifier and convolutional classifiers trained from scratch (F1 score 0.77–0.82, AUC 0.83–0.85). Occlusion sensitivity mapping in the correctly-classified cases (n = 160 PD and n = 114 PPS) highlighted the brain regions guiding discrimination mainly in sensorimotor and midline structures including cerebellum, brain stem, ventricle and basal ganglia.

**Interpretation:**

Our self-supervised digital model based on routine clinical head MRI discriminated PD vs PPS with good accuracy and sensitivity. With incremental improvements the approach may be diagnostically useful in early disease.

**Funding:**

10.13039/501100012166National Key Research and Development Program of China.

**Research in context**

**Evidence before this study**

Parkinsonian syndromes are a heterogeneous group of movement disorders that are challenging to early diagnostic differentiation in a clinical setting, because they often share many clinical features. There has been some research progress with tissue- and biofluid-based biomarkers, but these have disadvantages of accessibility, invasiveness or preanalytical and analytical confounders. We searched PubMed for original articles published in English, with the terms ‘Parkinsonism’, ‘Parkinson’s disease’, ‘Parkinson-plus syndrome’ combined with ‘MRI’. Previous studies have shown that MRI might be used to distinguish Parkinsonian syndromes, but they are typically conducted by highly-trained staff in specialised MR research centres, using high-resolution scanning protocols with stringent technical standards, involving lengthy data acquisition and complicated analysis. A screening model which could match this diagnostic performance using routine clinical MRI protocols would represent a significant advance for real-world clinical use. Using clinical head MRI scans with self-supervised learning offers unique clinical importance, because it reduces the reliance on large quantities of human annotated data, being trainable with fewer labelled examples due to faster convergence, and can be used on independent-sample testing to reach or exceed the performance of models trained and tested through supervised learning.

**Added value of this study**

In this study, we developed a pre-trained foundation model based on 75,861 clinical MRI scans across 14 MRI scanners for automated detection of n = 1992 participants with Parkinson’s disease and n = 1989 participants with Parkinson-plus syndrome, making this the largest clinical cohort of parkinsonism evaluated to date. The inputs were whole-brain images from a clinical routine protocol. Using a fully automated approach, we found that routine clinical head MRI is capable of differentiating Parkinson’s disease from Parkinson-plus syndrome, with high accuracy and interpretability. The regions contributing significantly to the classification were those previously shown to be pathologically involved in Parkinson’s disease and Parkinson-plus syndrome. This study developed a self-supervised digital model based on routine clinical head MRI data discriminated Parkinson’s disease and Parkinson-plus syndrome with good accuracy and sensitivity, which will expedite future studies in parkinsonism.

**Implications of all the available evidence**

This study provides an objective, validated, and generalisable imaging approach to distinguish Parkinsonian syndromes using clinical multi-contrast whole brain MRI. Our results are relevant in the clinical setting because they indicate that relatively lower resolution clinical MRI imaging could provide a screening model for physicians to use in considering a patient to have Parkinson’s disease or Parkinson-plus syndrome in a real-world clinical use. The outcome of this study suggests that the imaging and machine learning model might function well using data from different scanners.

## Introduction

Parkinsonian syndromes are a heterogeneous group of movement disorders. The most common is idiopathic Parkinson’s disease (PD), in which abnormal aggregates of alfa-synuclein are found in Lewy bodies and Lewy neurites. A clinical challenge, even for specialists, is early diagnostic differentiation of PD from Parkinson-plus syndrome (PPS),^1^ which share many clinical features; in addition to classical parkinsonian features, PPS have distinct aetiology and clinical features, poorer response to dopaminergic therapy, with a generally poorer prognosis. However, it is difficult to distinguish PPS from PD, especially in early stages: currently, diagnostic accuracy in early PD is 58%, and 54% of misdiagnosed patients have PPS.^2^^,^^3^ Accurate early diagnosis is important for management, and has considerable potential to improve disease progression and prognosis.^4^ There has been some research progress with tissue- and biofluid-based biomarkers, but these have disadvantages of accessibility, invasiveness or preanalytical and analytical confounders.^5^ An objective, reliable, non-invasive biomarker to distinguish Parkinsonian syndromes would be a step forward. Magnetic resonance imaging (MRI) offers such potential. Studies using MRI techniques such as diffusion, functional, and high-resolution structural imaging have reported high diagnostic accuracies (74–92%).^3^^,^^4^^,^^6^, ^7^, ^8^ However, such studies have limited practical application, being typically conducted by highly-trained staff in specialised MR research centres, using high-resolution scanning protocols with stringent technical standards, involving lengthy data acquisition and complicated analysis. A screening model which could match this diagnostic performance using routine clinical MRI protocols would represent a significant advance for real-world clinical use.

Unlike the typically well-defined, segmentable lesions of neoplastic disease, brain changes in Parkinsonian syndromes often lack distinct boundaries, necessitating examination of the whole brain. Deep learning-based algorithms outperform traditional feature engineering-based machine learning methods in analysing whole-brain images, automatically learning informative features without complex image processing.^9^, ^10^, ^11^ Studies using convolutional neural network (CNN) models to classify PD vs healthy controls have reported high accuracy.^12^, ^13^, ^14^, ^15^, ^16^, ^17^, ^18^, ^19^, ^20^, ^21^, ^22^ However, they typically sacrifice comprehensive image information (for example by focussing on regions of interest) to reduce the dimensionality of the individual data, and their classification efficiency is vulnerable to segmentation errors.^23^ One novel goal of our study is to overcome this limitation by using whole-brain images from a clinical routine protocol as inputs without relying on hand-selected features. Additionally, these learning-based models typically need large expert-labelled training datasets, which are time-consuming and cost-prohibitive to curate for medical images^24^ and often lead to overfitting, limiting their generalisability in clinical practice. The recently proposed method of self-supervised learning alleviates this by reducing the reliance on large quantities of human annotated data, being trainable with fewer labelled examples due to faster convergence, and can be used on independent-sample testing to reach or exceed the performance of models trained and tested through supervised learning.^24^^,^^25^ Self-supervised learning has been applied to research in natural language processing (NLP), computer vision, and medical data analysis,^24^^,^^26^^,^^27^ where labelled data are particularly scarce or expensive.^28^ It has not so far been used to develop MRI-based diagnosis of Parkinsonian syndromes, and this is what we set out to do.

**Fig. 1:**
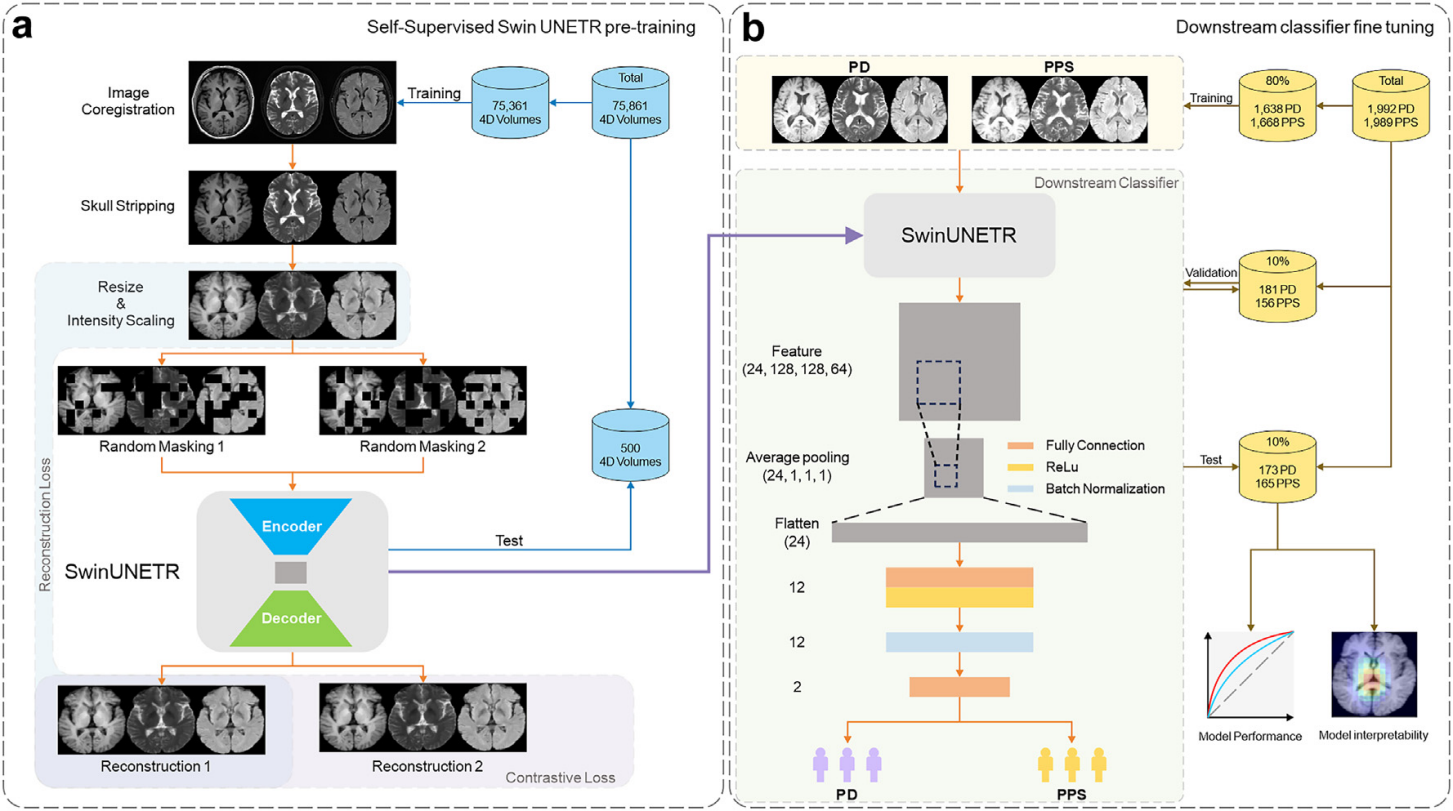
The flowchart of the training pipeline. Part (a) shows the pipeline for self-supervised Swin UNETR training, and Part (b) shows the pipeline for downstream classifier training. Data obtained from T1WI, T2WI, and FLAIR sequences were preprocessed to form a self-supervised pretraining dataset, finally incorporating 75,861 3-channel volumes from 14 MRI scanners. The downstream task involved the preprocessed images derived from 1992 patients with PD and 1989 with PPS. Abbreviations: PD, Parkinson’s disease; PPS, Parkinson-plus syndrome; FC, fully connected, ReLU, rectified linear unit.

The Masked Autoencoder (MAE) is an effective self-supervised learning strategy in computer vision^29^: portions of the image are masked, and the network is trained to recover the masked portion, thereby learning essential features of the image which can be used for downstream tasks like image classification. Using the Shift Window UNET TRansformer (Swin UNETR) approach, we developed a self-supervised learning framework (‘SwinClassifier’) to train an automatic PD vs PPS detection model in a large retrospective dataset acquired on several different MRI scanners in routine clinical conditions (whole brain T1WI, T2WI, and FLAIR) (Fig. 1). For comparison purposes we developed a comparative foundation model (‘ViTClassifier’) based on a vanilla Vision Transformer (ViT) autoencoder. Having tuned these foundation models, we evaluated them across downstream classifier tasks associated with the differential diagnosis of Parkinsonian syndromes. We also compared their performance with the CNN network models DenseNet121 and ResNet50, trained from scratch.

## Methods

### Ethical approval

This study conformed to the Helsinki Declaration and was approved by the ethics committee of Institutional Review Board (IRB) of West China Hospital, Sichuan University (reference number: 2019 (206) and 2023 (45)). Given the retrospective nature of the study, the IRB at the West China Hospital, Sichuan University reviewed the collection of participants data and creation of this research database and granted a waiver of informed consent.

### Participants

#### Task dataset

The task dataset, used for downstream classification, comprised MRI scans of n = 1992 participants with PD and n = 1989 participants with PPS retrospectively collected from the neurological inpatient clinics of West China Hospital, Sichuan University from December 2008 to July 2023. For PPS, the commonest diagnoses were multiple system atrophy (MSA) and progressive supranuclear palsy (PSP), but also included corticobasal degeneration and Lewy body dementia. Each patient’s diagnosis was obtained from hospital medical records, determined by movement disorder specialists according to published consensus criteria.^30^ These MRI scans were routinely performed in clinical settings to detect intracranial lesions e.g. neoplasm, stroke, haemorrhage, and infection. In patients having multiple MRI scans, the first scan suitable for analysis was selected. Exclusion criteria for all participants, based on medical records and radiological images, were: a history of stroke, brain trauma, or brain surgery; a history of neurological or psychiatric disease other than PD and PPS; any other degenerative disease; secondary Parkinsonism; treatment with deep brain stimulation; and poor imaging quality (e.g. head motion and artifact).

#### Pretraining dataset

The pretraining dataset used for the development of the self-supervised vision foundation model comprised plain head MRI scans obtained at our institution between January 2015 and December 2022, sourced from the Picture Archiving and Communication System (PACS). Scans from conditions resulting from artificial medical interventions or severe traumatic events, including post-surgical, post-radiotherapy, and severe traumatic brain injury, were excluded based on radiological reports and medical records. Additionally excluded were scans from PD or PPS patients included in the downstream classification model development. After quality check (see details in the Data Preprocessing section), a total of 75,861 scans from 71,239 participants (34,234 male, aged from 1 to 109 years, median age 53 years) acquired across 14 MRI scanners (details of scanners and basic sequence parameters are provided in Table 1) were included in self-supervised pre-training dataset. Demographics of these two datasets are presented in Table 2.

**Table 1:** MRI instruments, imaging sequences, and number of datasets acquired.

ID	Manufacturer	Model	Field strength	Imaging sequences and parameters		N
1	GE	SIGNA Architect	3 T	T1w	TR: 2196 ms TE: 6.5 ms R: 0.47 × 0.47 × 5 mm	8354
				T2w	TR: 5549 ms TE: 104 ms R: 0.47 × 0.47 × 5 mm	
				FLAIR	TR: 8500 ms TE: 94 ms R: 0.47 × 0.47 × 5 mm	
2	GE	SIGNA Premier	3 T	T1w	TR: 2417 ms TE: 6.3 ms R: 0.47 × 0.47 × 5 mm	7027
				T2w	TR: 5460 ms TE: 102 ms R: 0.47 × 0.47 × 5 mm	
				FLAIR	TR: 6500 ms TE: 91 ms R: 0.47 × 0.47 × 5 mm	
3	GE	Discovery MR750w	3 T	T1w	TR: 2993 ms TE: 23.5 ms R: 0.47 × 0.47 × 5 mm	14,513
				T2w	TR: 4921 ms TE: 94 ms R: 0.47 × 0.47 × 5 mm	
				FLAIR	TR: 8000 ms TE: 95.2 ms R: 0.47 × 0.47 × 5 mm	
4	GE	SIGNA Explorer	1.5 T	T1w	TR: 1663 ms TE: 30.8 ms R: 0.49 × 0.49 × 5 mm	2256
				T2w	TR: 5104 ms TE: 97 ms R: 0.49 × 0.49 × 5 mm	
				FLAIR	TR: 8000 ms TE: 118 ms R: 0.49 × 0.49 × 5 mm	
5	GE	SIGNA Excite	3 T	T1w	TR: 2367 ms TE: 27.6 ms R: 0.47 × 0.47 × 6 mm	919
				T2w	TR: 4000 ms TE: 106 ms R: 0.47 × 0.47 × 6 mm	
				FLAIR	TR: 7502 ms TE: 147 ms R: 0.47 × 0.47 × 6 mm	
6	Siemens	Skyra	3 T	T1w	TR: 1600 ms TE: 8.6 ms R: 0.86 × 0.86 × 5 mm	13,512
				T2w	TR: 4500 ms TE: 105 ms R: 0.5 × 0.5 × 5 mm	
				FLAIR	TR: 6000 ms TE: 81 ms R: 0.69 × 0.69 × 5 mm	
7	Siemens	Sonata	1.5 T	T1w	TR: 3100 ms TE: 11 ms R: 0.49 × 0.49 × 6 mm	1493
				T2w	TR: 3870 ms TE: 107 ms R: 0.45 × 0.45 × 6 mm	
				FLAIR	TR: 9740 ms TE: 92 ms R: 0.49 × 0.49 × 6 mm	
8	Siemens	Avanto	1.5 T	T1w	TR: 400 ms TE: 11 ms R: 1.2 × 1.2 × 6.5 mm	3113
				T2w	TR: 4200 ms TE: 85 ms R: 0.7 × 0.7 × 5 mm	
				FLAIR	TR: 6000 ms TE: 87 ms R: 0.9 × 0.9 × 5 mm	
9	Siemens	Trio Tim	3 T	T1w	TR: 1600 ms TE: 9.2 ms R: 0.78 × 0.78 × 5 mm	6697
				T2w	TR: 4000 ms TE: 93 ms R: 0.34 × 0.34 × 5 mm	
				FLAIR	TR: 6000 ms TE: 93 ms R: 0.43 × 0.43 × 5 mm	
10	Philips	Achieva	3 T	T1w	TR: 2000 ms TE: 10 ms R: 0.45 × 0.45 × 6 mm	4350
				T2w	TR: 3000 ms TE: 80 ms R: 0.43 × 0.43 × 6 mm	
				FLAIR	TR: 9000 ms TE: 120 ms R: 0.45 × 0.45 × 6 mm	
11	Philips	Ingenia Elition X	3 T	T1w	TR: 2000 ms TE: 20 ms R: 0.45 × 0.45 × 5 mm	4573
				T2w	TR: 3000 ms TE: 100 ms R: 0.4 × 0.4 × 5 mm	
				FLAIR	TR: 6900 ms TE: 150 ms R: 0.48 × 0.48 × 5 mm	
12	United Imaging	uMR588	1.5 T	T1w	TR: 2000 ms TE: 11.8 ms R: 0.6 × 0.6 × 6 mm	2556
				T2w	TR: 4500 ms TE: 90 ms R: 0.48 × 0.48 × 6 mm	
				FLAIR	TR: 8000 ms TE: 109 ms R: 0.6 × 0.6 × 6 mm	
13	United Imaging	uMR 790	3 T	T1w	TR: 832 ms TE: 9.7 ms R: 0.48 × 0.48 × 5.3 mm	3428
				T2w	TR: 5400 ms TE: 113 ms R: 0.46 × 0.46 × 5.5 mm	
				FLAIR	TR: 8000 ms TE: 102 ms R: 0.6 × 0.6 × 5.5 mm	
14	Toshiba	MRT200SP5	1.5 T	T1w	TR: 1900 ms TE: 15 ms R: 0.375 × 0.375 × 6 mm	3070
				T2w	TR: 4064.5 ms TE: 105 ms R: 0.375 × 0.375 × 6.2 mm	
				FLAIR	TR: 8600 ms TE: 105 ms R: 0.75 × 0.75 × 6.2 mm	

Abbreviations: FLAIR, fluid-attenuated inversion recovery image; ID, study scanner number; N, number of patient datasets acquired; R, in-plane spatial resolution; T1w, T1-weighted image; T2w, T2-weighted image; TE, echo time; TR, repetition time.

**Table 2:** Demographic characteristics of all participants from the pre-training dataset and the downstream classification dataset.

	Pre-training dataset	Downstream classification dataset	
	75,861 scans from 71,239 participants	PD (n = 1992)	PPS (n = 1989)
Age (y, mean ± SD)	50.4 ± 18.3	65.5 ± 11.8	63.8 ± 10.2
Sex n (%)			
Male	34,234 (48.1%)	1075 (54.0%)	1154 (58.0%)
Female	37,005 (51.9%)	917 (46.0%)	835 (42.0%)
Race/ethnicity n (%)			
Asian	71,239 (100%)	1992 (100%)	1989 (100%)
White	0	0	0
Black	0	0	0
Other	0	0	0

Abbreviations: y, years; SD, standard deviation; PD, Parkinson’s disease; PPS, Parkinson-plus syndrome.

### Data acquisition

All participants were scanned with clinical whole-brain MRI protocols including T1-weighted, T2-weighted and fluid attenuated inversion recovery (FLAIR) imaging. Scanning protocols for the pretraining dataset (75,861 clinical head MRI scans) are provided in the Table 1.^31^ In summary, the mean scanning time was approximately 4 min (with a range from 3 min 5 s to 5 min 30 s), slice thickness varied from 1 to 6.5 mm and in-plane image resolution from 0.34 × 0.34 to 1.2 × 1.2 mm^2^. The brain images of all participants were inspected by two radiologists (X.S. and L.C with 10 and 3 years of neuroradiologic MRI experience, respectively) to exclude any with structural brain abnormalities.

### Data sorting and preprocessing

All DICOM files were converted into Neuroimaging Informatics Technology Initiative (NIFTI) format (.nii). An in-house deep neural model automatically identified T1WI, T2WI and FLAIR volumes, and excluded images compromised by severe head motion, metal artifact and other imaging artifacts, and age artifacts.^32^ A whole-brain mask was generated by a skull stripping tool performed on T1WI to remove non-brain tissue using the HD-BET brain extraction tool.^33^ T2WI and FLAIR in the same scan were co-registered to the T1WI volumes selected for skull stripping using rigid registration implemented in Advanced Normalisation Tools (ANTs) and then multiplied by the brain mask. The preprocessed T1WI, T2WI and FLAIR images were then concatenated into a 3-channel 4D volume.

### Imaging processing procedure

Due to the high heterogeneity in scanning devices and protocols, a self-adaptive procedure was implemented to preprocess the raw data while preserving maximum information. The procedure follows this conditional logic:

1. High Resolution 3D T1 Weighted Volume (3D-T1WI) Available:
  o The 3D-T1 images are resampled to a 1 mm isotropic resolution.
  o Skull stripping is performed on 3D-T1WI.
  o Other contrasts are linearly co-registered to the resampled 3D-T1, then multiplied with the brain mask generated during skull stripping.
  o The co-registered volumes are concatenated into a single 4D volume in the order T1WI, T2WI, and FLAIR.
2. Only 2D Volumes Available:
  o The in-plane resolution of 2D-T1WI images is resampled to 0.8 × 0.8 mm to account for automatic in-plane interpolation by some scanners.
  o Skull stripping is performed.
  o Other contrasts are linearly co-registered to the resampled 2D-T1WI, then multiplied with the brain mask generated during skull stripping.
  o The co-registered volumes are concatenated into a single 4D volume in the order T1WI, T2WI, and FLAIR.

### Software and computer specifications

o **Skull stripping**: HD-BET (https://github.com/MIC-DKFZ/HD-BET) with default setting performed on a server equipped 8× NVIDIA RTX4090 GPU.
o **Co-registration**: antsaffine. sh script from ANTs package (https://github.com/ANTsX/ANTs) with default setting performed on a server equipped 2× AMD EPYC 7763 CPU (64 core and 128 threads for each CPU).
o **Mask multiplication and volume concatenation**: fslmaths tool from fsl package (https://fsl.fmrib.ox.ac.uk) with “-mul” option and fslmerge tool with “-t” option performed on a server equipped 2× AMD EPYC 7763 CPU (64 core and 128 threads for each CPU).

### Developing the self-supervised learning model

The Shift Window UNET TRansformer (Swin UNETR) served as the backbone for the development of the ‘foundation’ model, by which is meant a pretrained model capable of extracting image features (a ‘feature extractor’) and serving as a foundation for downstream task fine-tuning. The Swin UNETR is implemented using PyTorch and MONAI. The model was developed with a feature size of C (C = 24, depths: number of layers in each stage = (2, 2, 2, 2); num_heads: number of attention heads = (3, 6, 12, 24)). Each channel of the input 4D volume was resized to 128 × 128 × 64 and signal intensities within the channel were normalised to the range 0–1. In order to enable the deep neural network to implicitly learn the complementary relationship between the contrasts, different masking schemes are used for each channel. This approach allows for the recovery of the masked region to be based not only on the context within the channel, but also on the context across channels. The masking scheme, which consists of 84 randomly distributed masking blocks with dimensions of 16 × 16 × 16, collectively masked approximately 40% of the content of each channel.

The volumetric data was fed into the encoder to produce encoded representations with a shape of $\frac{128}{2^{i}}\times \frac{128}{2^{i}}\times \frac{64}{2^{i}}\times 2^{\left(j-1\right)}C$ (where i, j∈{0,1,2,3,4,5}, and j is incremented by 1 if j = 0). The extracted feature maps were subsequently passed to a CNN-based decoder via skip connections at each resolution, following a U-shaped network design. The final reconstructed outputs were obtained using a 1 × 1 × 1 convolutional layer followed by a sigmoid activation function.

The loss function for foundation model training is a hybrid function that encompasses both reconstruction loss and contrastive loss: we used the L1 loss, defined as the mean absolute error between corresponding voxels in the original and reconstructed volumes, as the reconstruction loss, L_recon_; the contrastive loss L_contrast_ was devised to quantify the discrepancy between a pair of reconstructions from the same sample but different masking schemes. To mitigate the risk of vanishing or exploding gradients, the total loss is defined as L_total_ = L_recon_ × (1 + L_contrast_). The quality of image reconstruction was quantitatively evaluated using the structural similarity index measure (SSIM) and the peak signal-to-noise ratio (PSNR). The entire workflow of self-supervised foundation model development is shown in Fig. 1a.

### Experimental implementation

#### Foundation model implementation

The foundation model was implemented and trained using the PyTorch library^34^ (https://pytorch.org/) and the MONAI package^35^ (Medical Open Network for Artificial Intelligence, https://monai.io/). A total of 500 volumes from 500 different participants were randomly selected from the pre-training dataset for testing purposes, the remaining volumes (75,861–500 = 75,361) being used for model training in order to avoid potential exposure of the test data to the training process. The foundation model was trained on a server with 8 NVIDIA A100 Graphics Processing Units (GPUs) with data parallel. Hyperparameters for model training were chosen empirically based on dataset size and observed convergence behaviour. Adam optimiser and a fixed learning rate (1e-4) were employed to accelerate convergence, with performance on the test set evaluated at intervals of 5 epochs. Training lasted for 500 epochs after test loss plateaued around epoch 200, ensuring stability, and the network weights corresponding to the best performance were saved.

#### Developing the parkinsonian syndromes discrimination model

The Parkinsonian syndromes dataset (subject to the above-mentioned exclusion criteria) was randomly split into training, validation, and test sets with a ratio of 8:1:1. Table 3 summarizes the quantity of data used for model training, validation and testing in each task. SwinClassifier was trained on the training data utilizing cross entropy loss and AdamW optimiser.^36^ In downstream task where the availability of labelled data is limited, a cosine learning rate decay strategy was employed, with the initial learning rate set to 1e-5 and gradually reduced to 1e-9 over the course of the training. Training lasted for 200 epochs, with validation loss convergence observed after 100 epochs. The classification performance on the validation set was monitored at each epoch, and the network weights corresponding to the highest classification accuracy were saved.

**Table 3:** Detailed number of images used for each task.

Tasks	Training	Validation	Test	Total
Pre-training	75,361	500	0	75,861
Downstream task				
Parkinson’s disease	1638	181	173	1992
Parkinson-plus syndrome	1668	156	165	1989

### Model evaluation

The model performance was evaluated on a test set based on five key metrics: F1 score, precision, sensitivity, specificity, accuracy, and the area under the Receiver Operating Characteristic (ROC) curve (AUC).

### Saliency map analysis

**Fig. 2:**
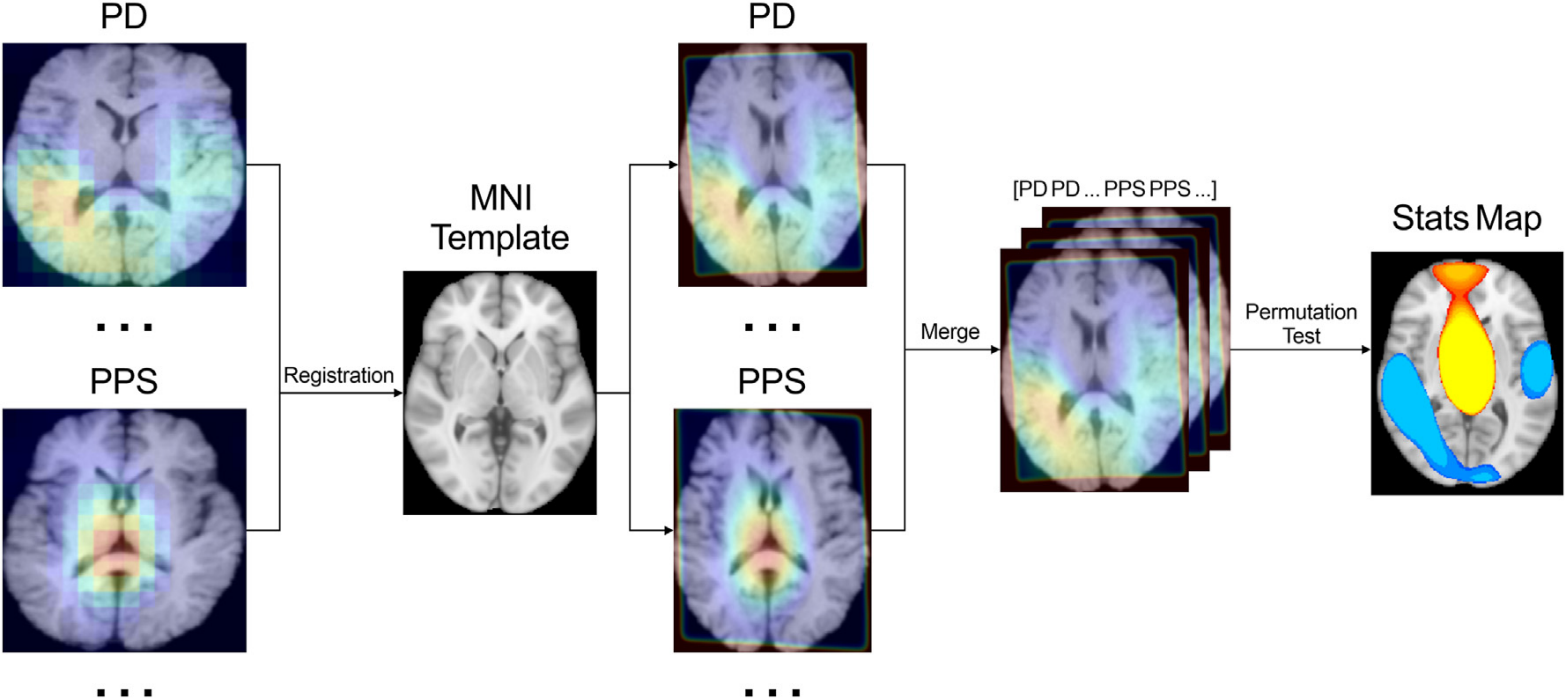
Workflow for generating statistical differences map of model-identified significant between-group discriminators. For each corrected classified subject in the test set, we generated saliency maps and registered both the subject’s T1 image and corresponding saliency maps to the Montreal Neurological Institute (MNI) standard space. We then spatially smoothed the saliency maps in MNI space. A general linear model was then applied to account for group-level differences, and voxel-wise statistical significance of the saliency maps was assessed using a permutation test. Abbreviations: PD, Parkinson’s disease; PPS, PPS, Parkinson-plus syndrome; MNI, Montreal Neurological Institute; Stats, statistical.

An occlusion sensitivity map was used to visualise the region of focus during the discrimination between PD and PPS. Furthermore, a voxel-wise analysis was conducted to investigate whether the identified regions differed significantly between the two groups (Fig. 2). Specifically, for each corrected classified subject in the test set, we generated saliency maps and registered both the subject’s T1 image and corresponding saliency maps to the Montreal Neurological Institute (MNI) standard space. We then spatially smoothed the saliency maps in MNI space. A general linear model was then applied to account for group-level differences, and voxel-wise statistical significance of the saliency maps was assessed using a permutation test.

### Model comparison

For comparative purpose we also developed a comparative foundation model based on a vanilla Vision Transformer (ViT) autoencoder.^31^ We also compared the foundation models’ performance with DenseNet121 and ResNet50, both CNN models trained from scratch.

### Statistics

To compare the performance of different models, the DeLong test was used to assess significant differences in AUC values across different models.

### Role of funders

The funders of the study had no role in study design, data collection, data analysis, data interpretation, or writing of the report. The corresponding author had full access to all the data in the study and had final responsibility for the decision to submit for publication.

## Results

### Reconstruction performance of the pre-trained foundation model

**Fig. 3:**
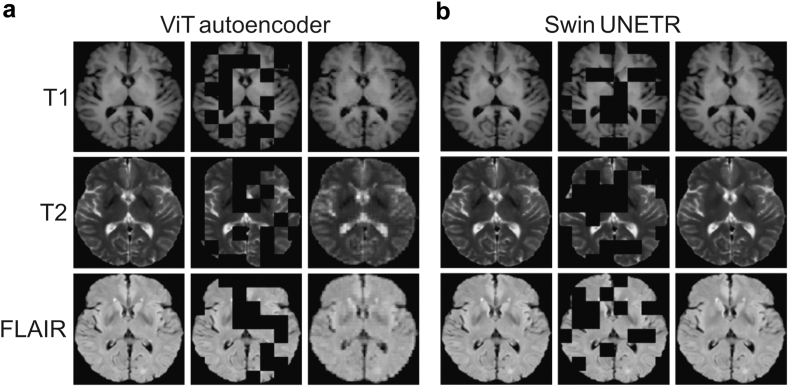
Reconstruction of a representative case from the pre-training dataset by the ViT autoencoder (a) and Swin UNETR (b). Abbreviations: T1, T1-weighted imaging; T2, T2-weighted imaging; FLAIR, fluid attenuated inversion recovery; Orig, origin; Recon, reconstruction.

The ViT autoencoder model achieved an SSIM of 0.87 ± 0.01 and a PSNR of 21.52 ± 0.70 (paired t-test P < 0.01), indicating moderate reconstruction quality and structural preservation. In contrast, the Swin UNETR model significantly outperformed ViT autoencoder model, attaining an SSIM of 0.97 ± 0.01 and a PSNR of 34.54 ± 1.19 (paired t-test P < 0.01), reflecting superior image reconstruction fidelity and perceptual quality. Fig. 3 shows the reconstruction results of the pre-trained ViT autoencoder and Swin UNETR.

### Model performance in PD vs PPS classification

**Fig. 4:**
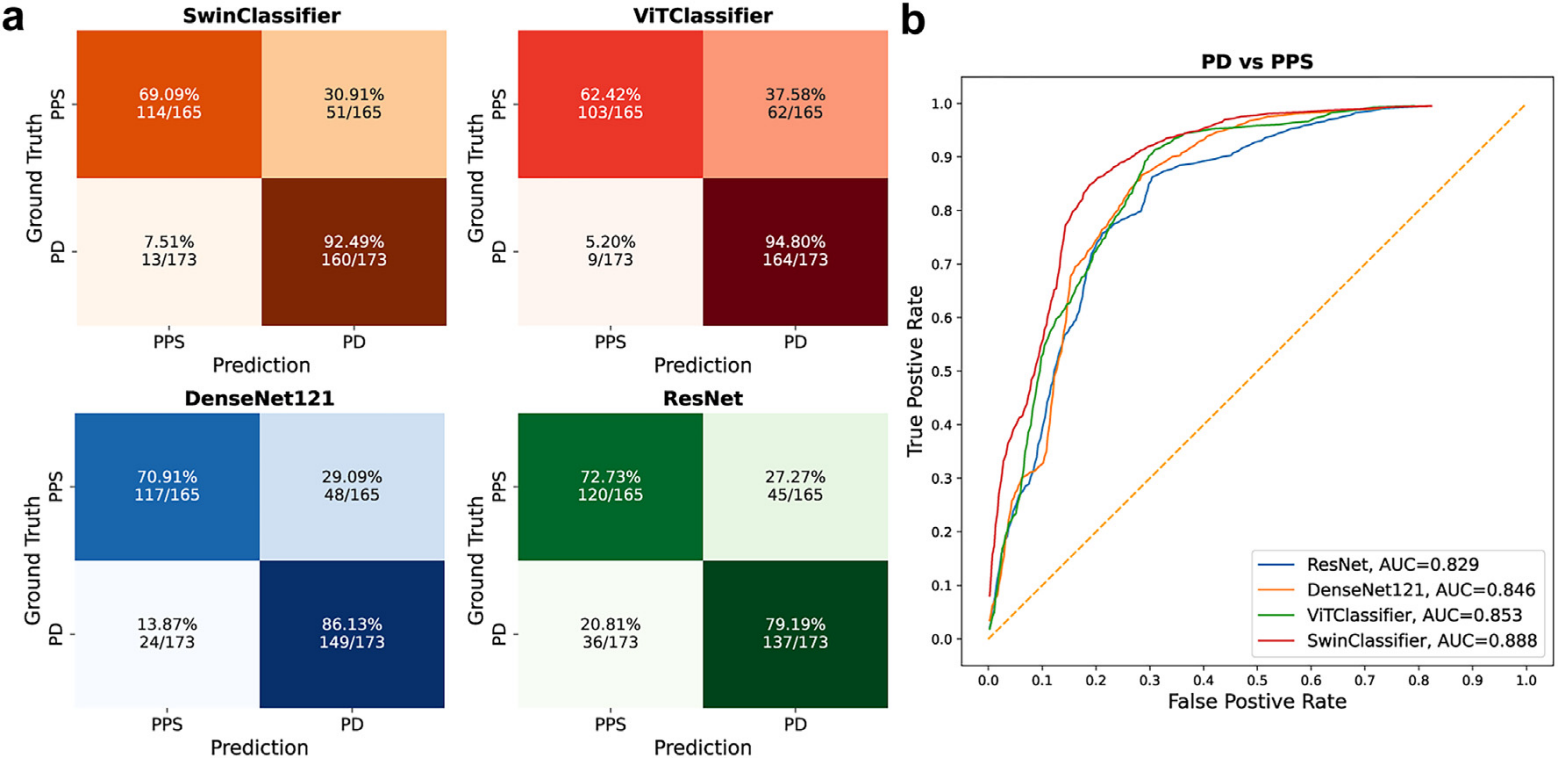
Performance of the classifiers. The figure shows the confusion matrices (a) and the corresponding ROC curves (b) for PD vs PPS discrimination on the test sets of the downstream tasks. Abbreviations: PD, Parkinson’s disease; PPS, Parkinson-plus syndrome; ROC, Receiver Operating Characteristic; AUC, area under the ROC curve.

We performed the downstream PD vs PPS classification task on an independent test dataset of n = 173 participants with PD and n = 165 participants with PPS, and the models’ classification efficacy on the test dataset was evaluated using a confusion matrix, as depicted in Fig. 4. SwinClassifier differentiated PD vs PPS with an accuracy of 81% (95% confidence interval [CI]: 77%–85%) with sensitivity 0.93 [95% CI: 0.88–0.96], specificity 0.69 [95% CI: 0.62–0.76], F1 score 0.83 [95% CI: 0.79–0.87], precision 0.76 [95% CI: 0.70–0.82], AUC 0.89 [95% CI: 0.85–0.92] (Table 4). This performance surpassed that of the comparative self-supervised foundation model ViTClassifier (accuracy 79% [95% CI: 75%–83%], sensitivity 0.95 [95% CI: 0.91–0.98], specificity 0.62 [95% CI: 0.55–0.70], F1 score 0.82 [95% CI: 0.78–0.86], precision 0.73 [95% CI: 0.67–0.78], AUC 0.85 [95% CI: 0.81–0.89]), and even more so DenseNet121 (accuracy 79% [95% CI: 75%–83%], sensitivity 0.86 [95% CI: 0.81–0.91], specificity 0.71 [95% CI: 0.64–0.78], F1 score 0.81 [95% CI: 0.76–0.84], precision 0.76 [95% CI: 0.69–0.81], AUC 0.85 [95% CI: 0.80–0.89]) and ResNet50 (accuracy 76% [95% CI: 72%–81%], sensitivity 0.79 [95% CI: 0.73–0.86], specificity 0.73 [95% CI: 0.67–0.79], F1 score 0.77 [95% CI: 0.72–0.82], precision 0.75 [95% CI: 0.69–0.81], AUC 0.83 [95% CI: 0.78–0.87]) models trained from scratch (Table 4).

**Table 4:** Performance of models with pre-trained weights and models trained from scratch.

Model	Accuracy (%, 95% CI)	Sensitivity (%, 95% CI)	Specificity (%, 95% CI)	Precision (95% CI)	F1 Score (95% CI)	AUC (95% CI)
Self-supervised foundation models with pre-trained weights						
SwinClassifier	274/338 (81.1%, 76.6%–85.2%)	160/173 (92.5%, 88.3%–96.3%)	114/165 (69.1%, 61.9%–76.1%)	0.758 (0.700–0.817)	0.833 (0.790–0.873)	0.888 (0.850–0.920)
ViTClassifier	267/338 (79.0%, 74.8%–83.1%)	164/173 (94.8%, 91.2%–97.8%)	103/165 (62.4%, 55.1%–69.9%)	0.726 (0.667–0.784)	0.822 (0.781–0.861)	0.853 (0.813–0.894)
Convolutional models strained from scratch						
DenseNet121	266/338 (78.7%, 74.6%–82.6%)	149/173 (86.1%, 80.8%–90.9%)	117/165 (70.9%, 64.1%–77.6%)	0.756 (0.694–0.810)	0.805 (0.761–0.844)	0.846 (0.804–0.885)
ResNet50	257/338 (76.0%, 71.6%–80.8%)	137/173 (79.2%, 72.9%–85.6%)	120/165 (72.7%, 66.5%–79.3%)	0.753 (0.691–0.810)	0.772 (0.721–0.819)	0.829 (0.784–0.870)

Abbreviations: Swin, Shift Window; ViT, Vision Transformer; AUC, area under the receiver operating characteristic curve; CI, confidence interval.

### Model comparison in PD vs PPS classification

We used the DeLong test for pairwise comparison of classification performance among the different models. The SwinClassifier demonstrated significantly superior classification performance compared to the DenseNet121 (P = 0.007), ResNet50 (P = 0.002), and ViTClassifier (P = 0.002) models. No statistically significant difference was observed in the following comparisons: ViTClassifier vs DenseNet121 (P = 0.81), ViTClassifier vs ResNet50 (P = 0.46), or DenseNet121 vs ResNet50 (P = 0.39).

### Saliency map of the swin UNETR model in PD vs PPS classification

**Fig. 5:**
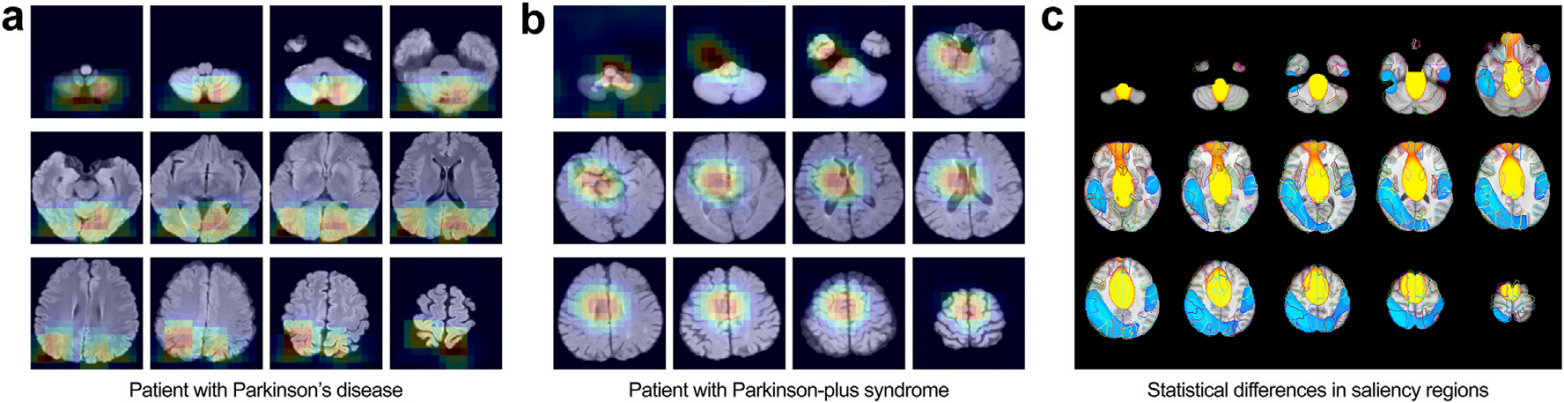
Representative saliency maps generated using occlusion sensitivity for correctly classified cases from the test sets of the downstream tasks. Warmer colours indicate regions with relative higher contribution weight in identifying PD or PPS. (a): saliency map of brain regions that contributed to the PD identification. (b): saliency map of brain regions that contributed to PPS identification. (c): voxel-wise statistical significance of the saliency maps between PD (blue) and PPS (yellow) with P < 0.05, threshold-free cluster enhancement (TFCE) corrected. Anatomical boundaries from the Automated Anatomical Labelling (AAL v3) atlas are overlaid as coloured borders. Abbreviations: PD, Parkinson’s disease; PPS, Parkinson-plus syndrome.

The saliency maps (Fig. 5a and b) show that in the correctly-classified cases (n = 160 participants with PD and n = 114 participants with PPS), the regions contributing significantly to the classification were mainly located in the sensorimotor cortex and midline structure including cerebellum, brain stem, ventricle, and basal ganglia. Furthermore, a voxel-wise analysis was conducted to investigate whether the identified regions differed significantly between the two groups (Fig. 2). As shown in Fig. 5c, the voxel-wise statistical analysis revealed differentially activated brain regions between the two groups (P < 0.05, threshold-free cluster enhancement corrected), with PD involved in posterior brain regions (including the parietal and temporal lobes) and PPS involving midline regions (including brain stem, basal ganglia, and ventricle), indicating that the main findings were consistently observed across MRIs.

## Discussion

To help fill the clinical need for noninvasive diagnosis of categories of Parkinsonian disease, we developed an SSL model (SwinClassifier) to automatically distinguish PD vs PPS, using MRI data obtained with both 3.0 T and 1.5 T scanners running standard clinical protocols, which offer faster acquisition and lower spatial resolution than typical in research studies. The performance of SwinClassifier surpassed that of ViTClassifier, a comparative foundation model which also used pretrained weights but with a different architecture, and even more so that of DenseNet121 and ResNet50, both trained from scratch. Notably, our classification accuracy was close to previous classification modelling studies using research data acquired using higher resolution scan protocols with longer acquisition time.^14^^,^^37^

SSL leverages large amounts of unlabelled data to learn generalisable feature representations.^25^ Rather than requiring human-annotated labels, the model creates ‘pretext tasks’ (e.g. predicting masked parts of an image or reconstructing missing data) that guide it to learn meaningful patterns inherent in the data.^24^ This approach contrasts with traditional supervised learning, which relies on manually labelled datasets and can be limited by the quantity and cost of expert-annotated samples–often a challenge in the clinical setting.^24^ Once pre-trained in a self-supervised manner, the model captures robust features that can be fine-tuned with relatively few labelled examples.^27^ This makes SSL particularly valuable for clinical applications where collecting large, high-quality, labelled datasets is time-consuming and expensive. By leveraging representations learnt from very large unlabelled datasets (e.g. medical images or electronic health records), SSL can improve performance on subsequent classification, detection, or prognosis tasks (i.e. ‘downstream tasks’) even when labelled data are scarce.^38^ Several studies have demonstrated the efficacy of SSL in medical imaging domains, such as fundus imaging,^25^ detection of pathologies from unannotated chest X-ray images,^26^ mammography classification, dermatology and pathology imaging,^38^ cancer-related diagnostic/prognostic imaging biomarkers,^39^ and brain disorder detection using functional MRI (fMRI).^40^ Our work extends this earlier research by constructing an imaging-aided model with cross-validation that could aid in detecting Parkinsonism-associated conditions using head MRI scans collected with clinical protocols, which might be useful in supplementing routine clinical practice.

While transformer architecture have revolutionised NLP, their adoption in medical imaging has been relatively limited. Recently, ViT has emerged as a powerful tool, achieving impressive results in medical imaging tasks such as image classification, object detection, and semantic segmentation. By adapting the scaling success of NLP transformers to images with minimal modifications, ViT have shown promise in applications like brain tumour^41^ and stroke lesion^42^ segmentation, brain age estimation,^43^ and the classification and registration of neurodegenerative diseases.^44^^,^^45^ fMRI, which provides more sensitive insights into cognitive decline and states–especially in early disease stages–can be incorporated as additional input channels in transformer models.^46^ However, current research relies on high-resolution scans from specialised MR centres equipped with high-field scanners, requiring lengthy acquisition times, often extending to dozens of minutes when combining fMRI with anatomical scans. This imposes significant costs and staff demands. In this study, we aimed to address these challenges by developing a screening model that achieves comparable diagnostic accuracy using much shorter scan protocols, offering a practical and scalable advance for real-world applications in neurological disease diagnosis and monitoring.

Major challenges in developing PD diagnostic AI models include architectural optimisation and generalisability. SwinClassifier addressed these, achieving 81% accuracy on T1/T2-weighted MRI data, within the 60–100% range reported in previous studies.^4^^,^^7^^,^^8^^,^^47^, ^48^, ^49^, ^50^, ^51^, ^52^ While reported accuracies close to 100% seem impressive, some of these studies used traditional radiomics strategies, some reports were not peer-reviewed, while some achieved high accuracies on very small and imbalanced datasets and/or region-of-interest-based features. This ‘result inflation’ is a recognised issue in neuroimaging research.^21^ In contrast, our machine learning models were developed using a much richer and balanced dataset of PD and PPS patients based on routine clinical MRI protocols, more closely resembling the real clinical world where collecting highly homogeneous data is essentially impossible. The model demonstrates strong generalisation to new data, as evidenced by its performance on a varied test set comprising data from multiple scanner manufacturers and field strengths. Our model represents a significant advancement in developing a computer-aided diagnosis tool for distinguishing PD from PPS, as it effectively identifies clinically relevant brain imaging differences despite data heterogeneity.

Furthermore, many studies use complex preprocessing pipelines to enhance deep neural network performance on specific datasets, such as using as input the preprocessed macro- and micro-structural metrics of brain tissues,^21^ or the segmented deep grey matter nuclei^53^; any shortcomings in these preprocessing steps risk erroneous predictions. In marked contrast, we adopted a strategy that inputs multi-contrast whole-brain volumes directly into the deep neural network for prediction, requiring minimal preprocessing and no brain segmentation. This eliminates segmentation failure risk and enhances the model’s clinical applicability.

Additionally, the diagnostic accuracy of AI models can differ across various MRI scanners and acquisition protocols, due to changes in imaging parameters that can impact image quality and features. We addressed issues related to differences in scanner manufacturer and field strength when constructing the pretrained foundation models. The pre-trained self-supervised foundation model can extract valuable representations from high-dimensional data, provide good initialisation values of network weights for the downstream task and avoid the occurrence of overfitting, which significantly reduces the demand for labelled data.

The brain areas we found to contribute most to PD vs PPS discrimination, being mainly in sensorimotor cortex and midline structures including cerebellum, brain stem, ventricle and basal ganglia, are consistent with existing understanding and in line with previous studies.^8^^,^^47^^,^^54^, ^55^, ^56^ In PD, persistent dopamine deficiency in the nigrostriatal pathway disrupts the interaction between the basal ganglia and motor cortex, resulting in the development of cardinal motor signs and symptoms.^57^ Unsurprisingly, alterations in the sensorimotor network are consistently reported in PD, and are an early predictor of disease severity.^57^ The cerebellum plays an important role in sensorimotor dysregulation and there is growing recognition of its contribution to motor and non-motor symptoms of PD.^58^^,^^59^ Patients with PSP exhibit significant midbrain atrophy (the ‘hummingbird sign’).^60^ Enlargement of the third ventricle is an MRI marker for early differentiation of PD vs PSP/MSA.^56^^,^^61^ Putaminal or pontocerebellar atrophy is reported in MSA and PSP, compared with PD.^56^ While the diagnostic sensitivity and specificity for differentiating PD vs PPS by assessing the midline brain area are typically high, the visual evaluation of this area remains qualitative, subjective, and heavily reliant on the physician’s expertise. Manual segmentation of brain MRI scans is arduous, time-consuming, and highly skilled work. Various automated brain structure segmentation methods have been developed,^3^^,^^8^^,^^62^ however, their use in clinical practice is restricted by their complexity and typically slow operation. Importantly, in early disease phases, clinically and in brain MRI, the diagnostic uncertainty is largest,^51^ because abnormalities may not yet have fully developed. In our SSL model, all these brain structure abnormalities contributed to the discrimination of PD vs PPS, including subtle changes largely inaccessible to the unassisted, even expert, naked eye. Taken together, the identified contributing regions for patient identification were consistent with established neuroimaging findings, supporting the promising clinical applicability of this model for disease detection.

Notably, the sensitivity of the Transformer-based model for PPS discrimination is significantly lower than that for PD and slightly lower than the CNN-based model for PPS discrimination. One possible explanation is that the lesion in PPS is focal and its alteration is subtle. While Swin UNET outperforms the traditional vision transformer in capturing fine-grained details, it may lack the inductive bias for local feature learning that CNNs possess, making it less effective for tasks requiring strong local feature extraction. In contrast, the lesions in PD are diffusely distributed, and Swin transformer excels at capturing long-range dependencies, enabling it to model global contextual relationships more effectively than CNNs. Although the Swin transformer strikes a balance between local and global feature learning (and thus achieves the best overall performance in this study), further enhancement and optimisation of the network architecture are needed to improve the learning and identification of features for different lesion types.

This work has limitations. First, participants with PD and PPS were clinically diagnosed, without pathology results; however, accurate diagnosis was supported by consensus operational clinical diagnostic criteria and 2 years average follow-up period. Second, the relatively small sample precluded analysis of different types of PPS; this will require larger studies. Third, we did not apply any additional harmonisation techniques to address potential scanner-specific biases besides intensity scaling. Without harmonisation, scanner-specific variations in scanner inherent characteristics and imaging parameters could introduce biases and potentially obscure the performance of our proposed classification model, despite the goal of our model to extract scanner-independent representations. Although our single-centre study included data acquired using multiple scanner models and field strengths, external validation with a multicentre dataset or a larger cohort will be important to confirm the robustness and generalisability of the findings. Finally, our study exclusively examined MRI-based diagnosis of PD and did not incorporate multi-modal data such as positron emission computed tomography, disease severity, comorbidities, medication use, or cerebrospinal fluid biomarkers. As a retrospective investigation, this study lacks prospective validation required to assess the real-world practical utility of mode-identified biomarkers. To bridge this gap, we share our foundation model and reproducible training pipeline to facilitate broader validation of their generalisability in future more comprehensive studies integrating diverse types of data.

Notwithstanding these limitations, in this study we developed a pre-trained foundation model based on an extensive corpus of clinical MRI scans for automated detection of patients with Parkinsonian syndromes that showed promising performance and interpretability. While neuroimaging research of Parkinsonian syndromes has appropriately, used images with high spatial and temporal resolution for more detailed mechanistic analysis, our study shows that an SSL model can effectively detect Parkinsonian syndromes using relatively lower resolution clinical imaging. Future work should focus on optimally integrating this type of model with other biomarkers, clinical assessment and observations to enhance earlier disease and risk determination by combining the AI models’ strengths with the clinician’s experience and judgement.

## Contributors

X.S. and H.S. conceived and designed the study. X.S., M.C. and H.S. have directly accessed and verified the underlying data reported in the manuscript. X.S., L.C., and C.L. collected the data. M.C. and H.S. analysed the data. X.S. draughted the manuscript. M.C., L.C., C.L., G.K., S.L., and H.S. contributed critical intellectual input to the manuscript. All authors approved the final version to be submitted for publication and agree to be accountable for all aspects of this work.

## Data sharing statement

The data that support the findings of this study are not publicly available due to privacy or ethical restrictions of West China Hospital of Sichuan University, but anonymized data are available on request from the corresponding author. The code and pretrained foundation models are available at https://github.com/MAI-Lab-West-China-Hospital/SwinBrain.

## Declaration of interests

The authors declare that they have no competing interests.
